# LIVERATION trial: a multicentre European randomised study on radiofrequency margin coagulation and its impact on oncological outcomes after liver surgery – study protocol

**DOI:** 10.1136/bmjopen-2025-100518

**Published:** 2025-11-24

**Authors:** Eduardo Luque Villalobos, Benedetto Ielpo, Luca Aldrighetti, Alessandro Anselmo, Nassiba Beghdadi, Giammauro Berardi, Francisco Briceño, Ruben Ciria, Dimitri Dorcaratto, Adam Durczynski, Giuseppe Maria Ettorre, Antonella Delvecchio, Valentina Ferri, Michał Grąt, Marina Garces-Albir, Lukasz Filip Grochola, Piotr Hogendorf, Francesco Izzo, Konrad Kobryn, Elissaios Kontis, Dimitris Korkolis, Robert Król, Mickael Lesurtel, Santiago Lopez-Ben, Christos Christogiannis, Ourania Koutsiouroumpa, Dimitris Mavridis, Riccardo Memeo, Dawid Murawa, Nikolaos Machairas, Isabel Mora-Oliver, Elena Muñoz-Forner, Carola Orrego, Carina Dantas, Carlos Fuste, Alberto García Picazo, Patrick Pessaux, Tomasz Pilat, Gabriella Pittau, Aleksandar Radosevic, Francesca Ratti, Georgios Sotiropoulos, Konstantinos Toutouzas, Blaz Trotovsek, Wojciech Wystrychowski, Dimitris Zacharoulis, Emilio Vicente, Fernando Burdio, Patricia Sánchez Velázquez

**Affiliations:** 1 Department of HBP Surgery, Hospital del Mar, Barcelona, Catalunya, Spain; 2 Hospital del Mar Research Institute IMIM, Barcelona, Catalunya, Spain; 3 Universitat Pompeu Fabra, Barcelona, Catalunya, Spain; 4 Department of HBP Surgery, IRCCS Ospedale San Raffaele, Milan, Lombardy, Italy; 5 Department of HBP Surgery, Fondazione PTV Policlinico Tor Vergata, Roma, Lazio, Italy; 6 Department of HBP Surgery and Liver Transplantation, Beaujon Hospital, Assistance Publique Hôpitaux de Paris, University of Paris Cité, Clichy, Île-de-France, France; 7 Department of HBP Surgery, San Camillo Forlanini Hospital, Roma, Lazio, Italy; 8 Department of HBP Surgery and Liver Transplantation, Hospital Universitario Reina Sofia, Córdoba, Andalucía, Spain; 9 Liver, Biliary and Pancreatic Unit. Department of General and Digestive Surgery, Hospital Clinico Universitario of Valencia. Department of Surgery. University of Valencia. Biomedical Research Institute, INCLIVA, Valencia, Spain; 10 Department of HBP Surgery, Norbert Barlicki Memorial University Hospital, Medical University of Lodz, Lodz, Poland; 11 Unit of Hepato-Biliary and Pancreatic Surgery, “F. Miulli” General Hospital, 70021 Acquaviva delle Fonti, Bari, Italy; 12 Department of Medicine and Surgery, LUM University, 70010 Casamassima, Bari, Italy; 13 Service of General and Digestive Surgery, Sanchinarro University Hospital, Clara Campal Oncological Center, Health Sciences Faculty, HM Hospitals. Carrillo José Cela University, Madrid, Community of Madrid, Spain; 14 Department of General, Transplant and Liver Surgery, Medical University of Warsaw, Warsaw, Poland; 15 Department of HBP Surgery, Kantonsspital Winterthur, Winterthur, Zurich, Switzerland; 16 Department of HBP Surgery, Istituto Nazionale Tumori IRCCS Fondazione Pascale, Naples, Campania, Italy; 17 Department of HBP Surgery, Metaxa Cancer Hospital of Piraeus, Piraeus, Attika, Greece; 18 Department of HBP Surgery, Agios Savvas General Cancer and Oncology Hospital of Athens, Athens, Attica, Greece; 19 Department of HBP Surgery, Medical University of Silesia in Katowice, Department of General, Vascular and Transplant Surgery, Katowice, Silesian Voivodeship, Poland; 20 Department of HBP Surgery, Hospital Universitari de Girona Doctor Josep Trueta, Girona, Catalunya, Spain; 21 Department of Primary Education, University of Ioannina, Ioannina, Greece; 22 Department of General Surgery and Surgical Oncology; Chair of Surgical Oncology, The Regional Hospital in Poznan –The Greater Poland Specialist Center; Collegium Medicum, University of Zielona Góra, Poznań, Zielona Gora, Poland; 23 Department of HBP Surgery, National and Kapodistrian University of Athens General Hospital of Athens Laiko, Athens, Attica, Greece; 24 Avedis Donabedian Instituto Universitario UAB, Barcelona, Spain; 25 Universitat Autònoma de Barcelona, Barcelona, Spain; 26 Research Network on Chronicity Primary Care and Prevention and Health Promotion, Madrid, Spain; 27 Shine 2Europe, Coimbra, Portugal; 28 University Hospital of Strasbourg, Department of HBP Surgery, Nouvel Hopital Civil, Strasbourg, France; 29 Department of HBP Surgery, Paul Brousse Hospital, Villejuif, Île-de-France, France; 30 Department of Radiology, Hospital del Mar, Barcelona, Catalunya, Spain; 31 Department of HBP Surgery, General Hospital of Athens Ippokrateio, Athens, Attica, Greece; 32 Department of Abdominal Surgery, University Medical Centre Ljubljana, Ljubljana, Slovenia; 33 Department of HBP Surgery, General University Hospital of Larissa, Larissa, Thessaly, Greece

**Keywords:** Hepatobiliary tumours, Hepatobiliary surgery, Surgical pathology, Carcinoma, Hepatocellular

## Abstract

**Introduction:**

Surgical margins are crucial in determining postoperative local recurrence (LR) in patients with colorectal liver metastasis (CRLM) and hepatocellular carcinoma (HCC). Achieving a margin greater than 1 cm can be challenging due to constraints related to remnant liver reserve, proximity to major vascular structures and tumour depth. We previously published findings from a retrospective study suggesting that additional margin coagulation (AMC) using radiofrequency may reduce LR, and this multicentre randomised clinical trial aims to further assess this hypothesis.

**Methods and analysis:**

The LIVERATION trial is an international, multicentre, single-blind, randomised, parallel-group, controlled clinical trial involving 698 patients undergoing liver resection for CRLM or HCC. Participants will be randomly assigned in a 1:1 ratio to either AMC (study group) or conventional liver resection (control group) to assess oncological outcomes for both CRLM and HCC. The primary outcome is the incidence of LR. Secondary endpoints include overall survival, disease-free survival, cancer-specific survival, surgical complications and quality of life. Follow-ups occur at 30 days, 90 days, and 1, 2 and 3 years postoperatively.

**Ethics and dissemination:**

The LIVERATION trial has been approved by the Ethics Committee at the sponsor site Hospital del Mar de Barcelona, CEIM-PSMAR (Comité de Ética de la Investigación con Medicamentos – Parc de Salut Mar), as well as by the Institutional Ethics Committees in all participating countries. The results of the main trial, along with each of the secondary endpoints, will be submitted for publication in a peer-reviewed journal. The study adheres to national and international guidelines, including the Declaration of Helsinki, and complies with regulations for studies involving biological samples under Law 14/2007 on Biomedical Research. A dissemination strategy has been developed to engage stakeholders and facilitate knowledge transfer to support the use of the findings of the study. LIVERATION is funded by the European Union under the Horizon Europe Framework Programme (Project Number: 101104360).

**Trial registration number:**

NCT05492136.

**STRENGTHS AND LIMITATIONS OF THIS STUDY**

- This study represents the first international randomised clinical trial specifically designed to evaluate the effect of additional coagulation of the resection margin after hepatectomy on local recurrence. Its pragmatic design ensures that the findings will be generalisable to routine clinical practice.
- The LIVERATION trial is endorsed by a Horizon Europe grant and involves collaboration with renowned high-volume liver surgery centres across Europe, guaranteeing both scientific rigour and broad clinical relevance.
- A potential limitation is the use of broad inclusion criteria, which, while increasing external validity, may also introduce heterogeneity and potential confounding factors that must be carefully addressed in the analysis.

## Introduction

 The liver is the primary site of metastasis for colorectal cancer,^1^ which is currently the third most common malignancy worldwide.^2^ Besides the recent advances in multimodal strategies for treating patients with colorectal liver metastasis (CRLM), there has been an increase in cure and/or durable relapse-free survival in patients with advanced disease, partly attributable to successful liver resections.^3^,^5^ The 5-year overall survival (OS) rate for colorectal cancer after hepatic resection is 25%–40%, compared with 0%–5% for those who will not have the surgery.^6^ Similarly, primary liver cancer is the sixth most commonly diagnosed cancer and the third leading cause of cancer death worldwide in 2020.^7^ As in CRLM, liver resection remains the standard curative treatment for hepatocellular carcinoma (HCC) in patients at early stages according to the Barcelona Clinic Liver Cancer classification (BCLC 0–A), with small solitary tumours and very well-preserved liver function.

While hepatic recurrence (HR) in the liver remnant appears to be more dependent on immutable tumour-related factors such as molecular pathology, biomarkers or initial size and tumour burden, local recurrence (LR) at the resection margin can be influenced by other modifiable factors.^8 9^ In line with this, surgical margin status has been considered to be of paramount importance for its impact on postoperative LR^10^; however, the appropriate width for a negative resection margin remains a topic of debate.^11^ Some consider a 1 cm margin as indicative of R0 status,^12^ while others accept subcentimetre negative margins, including those less than 1 mm, as R0.^11^ This distinction is even more important in the case of HCC, as these tumours are likely to present with satellitosis; thus, a wider margin is preferable compared with what is typically required for CRLM. Achieving a margin greater than 1 cm is clearly desirable and should be attempted whenever possible. However, this goal can be anatomically challenging due to factors such as insufficient future liver remnant, proximity to major vascular structures and tumour depth. Nonetheless, a questionable preoperative R0 status should not preclude a liver resection if it is feasible.

Radiofrequency (RF) ablation has gained popularity over the past two decades and has even challenged the indications for liver resection in specific cases of CRLM and HCC. Hepatobiliary surgeons have increasingly used energy-based sealing systems and haemostatic devices, such as RF monopolar electrodes, which not only facilitate bloodless liver resections^13 14^ but also appear to influence oncological outcomes by creating a substantial zone of thermally coagulated tissue at the transection line of the remnant liver.^15 16^ Given its established efficacy in tumour ablation, such as for HCC, RF is sometimes used to ablate the liver surface following questionable resections.

Despite the widespread use of additional coagulation in doubtful margins, high-level evidence supporting its effectiveness in reducing LR or improving OS is lacking. In a previous retrospective study, we investigated the concept of additional margin coagulation (AMC) during liver resections with narrow margins and found that the study group experienced significantly fewer LR compared with controls.^17^ Up to our knowledge, there are no clinical prospective randomised studies with the same scope as that of the LIVERATION trial. Therefore, the present study aims to further assess this approach through a European multicentre randomised clinical trial.

## Materials and methods

### Study design

LIVERATION is an international multicentre, single blind, randomised, parallel-group, controlled clinical trial conducted across seven European countries: Spain, France, Switzerland, Italy, Greece, Poland and Slovenia. The trial includes all consecutive patients scheduled for either conventional surgery or AMC of the transection surface to assess LR. Clinical sites participating in the study are academic hospitals across Europe, recognised for their expertise in liver surgery and strong academic backgrounds. Besides their recognised expertise in liver surgery, these centres are also high-volume institutions, each performing more than 50 liver procedures annually. The study is registered at ClinicalTrials.gov (NCT05492136) and is funded by the Horizon Europe Framework Programme (Project Number: 101104360) under the call Research and Innovation actions supporting the implementation of the Mission on Cancer https://ec.europa.eu/info/funding-tenders/opportunities/portal/screen/opportunities/topic-details/horizon-miss-2022-cancer-01-03.

This protocol was developed in accordance with the Standard Protocol Items: Recommendations for Interventional Trials (SPIRIT) guidelines.^18^ A completed SPIRIT checklist is provided in online supplemental file 1.

### Study population and eligibility criteria

All patients with CRLM or HCC will be assessed for eligibility for surgical resection by a multidisciplinary tumour board following current clinical practice. Patients who meet the inclusion criteria and do not meet any exclusion criteria will be approached by the investigators and recorded, regardless of whether they choose to participate. All patients will sign a written informed consent form (ICF) before randomisation.^18^ A copy of the ICF is available in the online supplemental material section.

Inclusion criteria:

- Written informed consent granted prior to the initiation of the surgical procedure, given with the understanding that the patient has the right to withdraw from the study at any time, without prejudice.
- Aged 18 years or older.
- WHO performance scale 0–2.
- Consecutive patients (both sexes equally distributed) suffering from CRLM confirmed either by abdominal CT, abdominal MRI and/or by histological–cytological evaluation, or patients suffering from HCC.
- Any previous chemotherapy regime is permitted.
- American Society of Anesthesiologists (ASA) score 1–3.

Exclusion criteria:

- Previous or concurrent cancer different from one primary tumour of which the liver metastasis comes from, except cervical carcinoma in situ, treated basal cell carcinoma and superficial bladder tumours (Ta, Tis and T1).
- Any cancer curatively treated >3 years prior to enrolment is permitted.
- American Society of Anesthesiologists (ASA) score 4.
- Non-resectable extrahepatic metastases.
- Liver metastasis from other origins apart from colorectal.
- Benign primary tumour of the liver.
- Pregnant woman.
- Participation in another clinical trial.

### Trial-specific interventions

The trial intervention will not influence the overall surgical procedure. Liver access will be achieved using the preferred approach of the surgeon—either open, laparoscopic or robotic—under general anaesthesia. The peritoneal cavity will be examined, and intraoperative ultrasonography will be performed as usual to detect any previously undetected lesions. If necessary, the liver will be mobilised according to the size and location of the lesion, and dissection of the hilar structures or hepatic veins is permitted in both study arms. After selecting the transection plane, resection of the liver will be performed using the technique chosen by the surgeon, following the standard clinical practice of each centre.

#### Arm 1 (control group)

Liver transection will be performed using conventional methods according to the preference of the surgeon, which may include one or more of the following techniques without specific coagulation of the margin after transection: conventional crush-clamp or finger fracture technique, ultrasonic dissector (eg, CUSA), ultrasonic-mediated devices, water jet dissector, argon beam coagulator or bipolar forceps.

#### Arm 2 (study group)

In this arm, the use of RF devices with evidence in the literature for reducing LR is permitted, eg, Coolingbis© (Vecmedical Spain, S.L. Montcada i Reixac, Catalunya, Spain) and Aquamantys© (Medtronic Advanced Energy, LLC. 180 International Drive, Portsmouth, New Hampshire, 03801, United States), either alone or in combination with any conventional method.

For Coolingbis® (5 mm model): after resection, the margin will be coagulated by moving the device over the tissue in a ‘painting motion’, covering 2.5 cm diameter circles for 15 s with the electrode tip at a power level of 7, ensuring complete surface coagulation (online supplemental video 1).

For Aquamantys®: the margin will be coagulated using a similar ‘painting motion’, covering 2.5 cm diameter circles for 15 s with the electrode tip at power of 100–120 W and medium saline flow. Continuous aspiration of hot saline will be performed during haemostasis to prevent thermal damage to surrounding tissues and organs.

Prior to the initiation of recruitment at each participating centre, a training and certification visit on the use of RF devices will be conducted, led by the study sponsor (IMIM). Follow-up and monitoring visits will be performed at each site to ensure proper use of the devices, as well as to verify appropriate calibration and quality control.

As the LIVERATION trial is pragmatic, no additional effort will be made to standardise postoperative care beyond ensuring that the same protocol is applied to both the AMC and control groups within each centre. Participants will receive postoperative care according to the routine practices of their respective centres. However, all surgical techniques, materials and medical devices used will be reported in detail to identify potential confounders and register any imbalances among treatment groups.

## Data capture and trial endpoints

*Primary endpoint(s)*: the primary endpoint of the study is LR, defined as any growing or enhancing tumour in the margin of hepatic resection^19^ specifically reviewed to this aim. LR will be assessed by follow-up imaging (CT or MRI) at 30 days (visit 3), 90 days (visit 4) and 1, 2 and 3 years after surgery (visits 5, 6 and 7, respectively).

Technical optimisation is essential to ensure high-quality (CT). Key parameters include: (i) multiphase acquisition with thin-section image reconstruction (≤3 mm slice thickness), comprising an arterial phase (20–25 s) of the upper abdomen and a portal venous phase (approximately 70 s) covering the abdomen and pelvis; (ii) administration of a non-ionic iodinated intravenous contrast agent at a dose of 1.5 mL/kg, injected at a rate of 4–5 mL/s.

Abdominal MRI is indicated when contrast-enhanced CT is contraindicated. In such cases, a non-contrast CT of the chest, abdomen and pelvis should also be performed. MRI protocols should include T1-weighted in-phase and out-phase, T2-weighted sequences with and without fat suppression, and diffusion-weighted sequences as well as multiphasic contrast-enhanced sequences. These parameters follow the recommendations from the European Society for Medical Oncology.^20^

All imaging will be interpreted locally by the reporting radiologist at each participating centre. Additionally, the scans will undergo central review by a designated radiologist (AR), who has 18 years of experience in pancreatic imaging and will be blinded to the surgical technique used.

Transection area (cm^2^): This will be obtained by delineating the transection plane from a digital photograph using dedicated software (3D Doctor, Able Software Corp, Lexington, MA, USA). To achieve this, a CT scan performed 1 month after surgery will be required. These images will be evaluated at the sponsor centre by an experienced radiologist specialised in RF ablation imaging.

*Secondary endpoint(s)*: Main secondary endpoints of the study will be OS, defined as time from surgery until death or the end of the study, patients who discontinue from the study without disease will be censored at the time of their last tumour evaluation prior to death or discontinuation. Disease-free survival (DFS), calculated as the time from surgery until disease progression, patients who have not progressed at the time the survival analysis review is performed will be censored at the time of their last tumour evaluation. Cancer-specific survival, measured from the date of surgery to the date of death from colorectal cancer/HCC or last follow-up. Hepatic recurrence (HR), calculated as the time from surgery until specific hepatic progression outside the resection margin.

Postoperative morbidity and in-hospital mortality will be assessed using Clavien-Dindo Classification^21^ and the Comprehensive Complication Index^22^ at 90 days. Index complications after hepatectomy were specifically recorded: postoperative hepatic failure was defined according to the ‘50–50 criteria’ on postoperative day 5. Biliary fistula was defined as total bilirubin level in drainage >3 times the level in serum or bile accumulation in the abdominal cavity.^23^ Other variables include patient baseline characteristics (ie, demographics, stage, date of diagnosis, tumour biomarker status and chemotherapy regime).

Quality of life (QoL) will be assessed using two patient-reported outcomes measures; the QLQ-C30,^24^ eligible for cancer patients in multicultural clinical research settings, including health, social and emotional domains; and the QLQ-HCC18^25^ which was developed as a supplement for patients with HCC. Both have been developed by the European Organisation for Research and Treatment of Cancer. Both questionnaires will be run at baseline visit and repeated every visit except for the day of surgery and the 90-day postoperative visit. We will also evaluate patient experience during cancer using one cancer-specific patient-reported experience measure to evaluate patient experience throughout the care pathway: an adapted version of the Swiss Cancer Patient Experiences questionnaire (V.2023); this questionnaire evaluates patient’ experiences of care from cancer diagnosis to follow-up care, in addition to evaluating overall satisfaction.^26^

Moreover, a socioeconomic analysis, grounded in the quality-adjusted life year framework, will be conducted alongside the clinical trial. This analysis will use data exclusively from the patients enrolled in the trial to estimate the broader economic impact and assess the cost-effectiveness of the treatments being evaluated with the final aim of informing healthcare policies, optimising resource allocation and justifying potential clinical interventions for patients affected by this condition. Primary and secondary outcomes can be checked in table 1.

**Table 1:** Study primary and secondary outcomes

Outcomes	Definition	Timeline
Primary		
Local recurrence	Growing or enhancing tumour in the margin of hepatic resection	Within 3 years from surgery
Secondary		
Overall survival	Time from surgery until death or the end of the study	Within 3 years from surgery
Disease-free survival	Time from surgery until disease progression	Within 3 years from surgery
Cancer-specific survival	Time of death from colorectal cancer/hepatocarcinoma or last follow-up	Within 3 years from surgery
Postoperative morbidity	Clavien-Dindo Classification and the Comprehensive Complication Index	Within 90 days from surgery
In-hospital mortality		Within surgery until hospital discharge
Quality of life questionnaires	Patient-reported outcomes measures: QLQ-C30^22^ and QLQ-HCC18^23^	Within 3 years from surgery
Experience questionnaire	Adapted version of the Swiss Cancer Patient Experiences^24^	Within 30 days from surgery

### Patient timeline and trial visits

**Figure 1:**
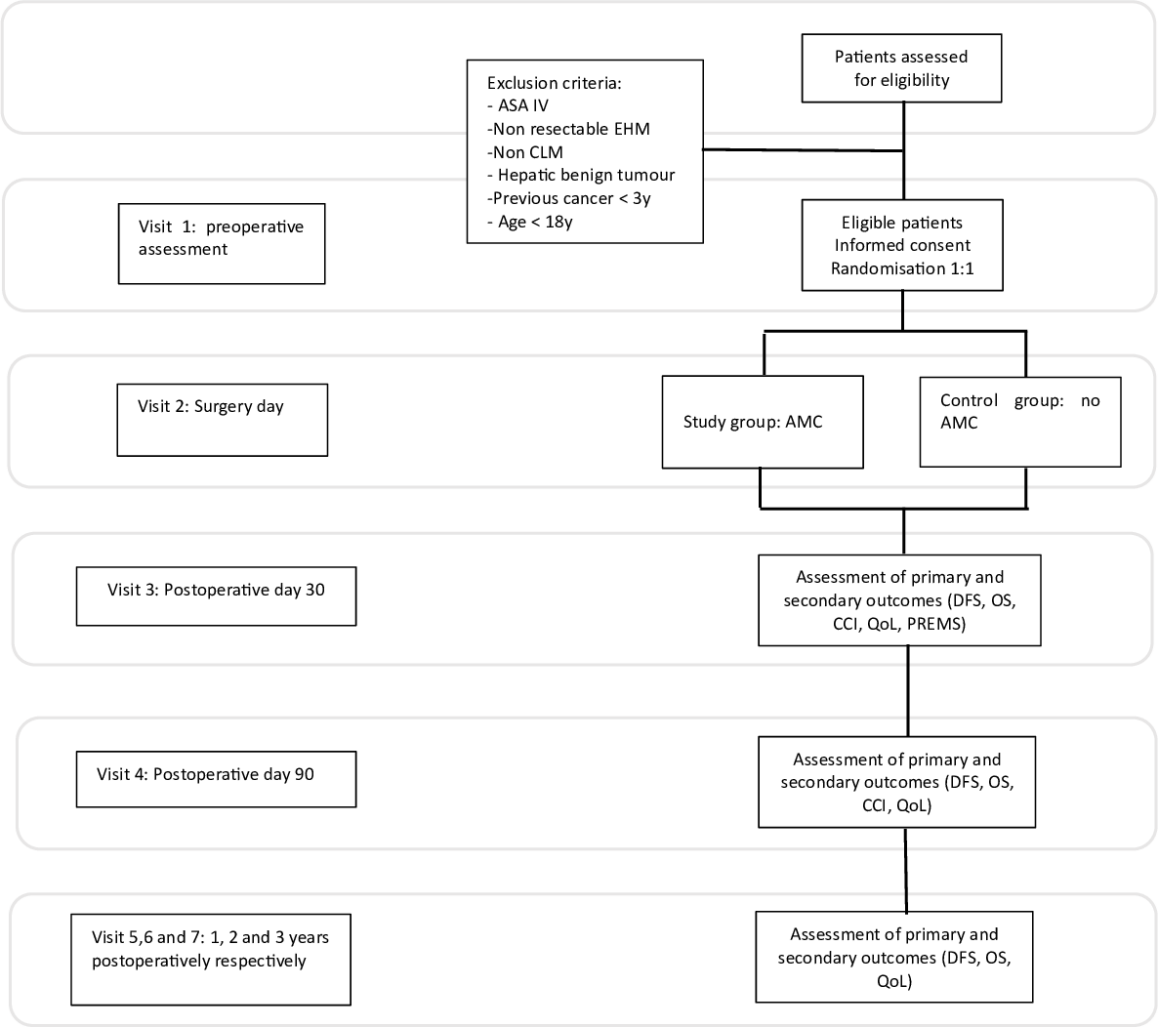
Patient flowchart. CCI, Comprehensive Complication Index; DFS, disease-free survival; OS, odds ratio; PREMs, patient-reported experience measure; QoL, quality of life; AMC, additional margin coagulation; CLM, colorectal liver metastasis; EHM, extrahepatic metastasis; ASA: American Society of Anesthesiologists.

All patients scheduled for elective surgery for CRLM or HCC in all the centres will be considered to participate in the trial and assessed for eligibility. Reasons for non-inclusion and those who refuse to participate must be documented. Patients will be enrolled based on their ability to comprehend the trial’s scope and nature and will provide written informed consent after receiving detailed information and meeting all inclusion criteria. Each patient must complete at least five follow-up visits (figure 1).

Baseline data, including routine blood tests, serum biochemistry, tumour markers, preoperative imaging (CT scan or MRI), and the initial QoL questionnaires, will be recorded during the baseline visit. Informed consent and randomisation will also occur during this first visit. Surgical data will be collected on visit 2, the day of surgery, which will be scheduled within 5 weeks after randomisation.

Primary and secondary outcome parameters will be collected during follow-up visits, specifically at 30 days (visit 3) and 90 days (visit 4) post-surgery. Subsequent follow-up visits (visits 5, 6 and 7) will be conducted at 1-year intervals (table 2).

**Table 2:** Trial visits and documented parameters

	Visit 1	Visit 2	Visit 3	Visit 4	Visit 5*	Visit 6*	Visit 7*
Assessment	Baseline	Surgery day	POD 30	POD 90	**1 year**	**2 years**	**3 years**
Eligibility assessment, informed consent and randomisation	X						
Medical history	X						
Physical examination	X		X	X	X	X	X
Surgery		X					
Blood analysis	X		X	X	X	X	X
Radiological imaging	X		X	X	X	X	X
QoL questionnaires†	X		X		X	X	X
PREMs questionnaire†			X				

* A window of ± 30 days will be taken into consideration for the completion of visits 5, 6 and 7.

† In the event that, due to some extraordinary circumstance, it is not possible to see the patient in person, it will be possible to call the patient by phone to administer the questionnaires telephonically (with their prior consent), no later than 1 month after the scheduled date for questionnaire administration. In the case of the quality of life questionnaires to be administered at visit 1, it is crucial to administer them prior to the surgery. The patient may also be contacted by phone in the event the investigator needs to clarify any questions or fill in unanswered questions.

POD, postoperative day; PREMs, patient-reported experience measures; QoL, quality of life.

### Calculation and justification of the sample size

Sample size calculation was done using Stata statistical software (StataCorp LLC, 4905 Lakeway Drive, College Station, Texas, 77845, United States) and the clustersampsi command V.st0286 2.^27^ We estimated that the event rate of LR in CRLM is 10.2% and 20% for HCC.^28^ At a significance level of 5% and assuming 10% attrition, we calculated that 518 CRLM participants would provide the trial with 80% power to detect an absolute between-group difference of 6.9% (reducing the risk of LR from 10.2% in the control group to 3.3% in the treatment group). Similarly, we calculated that 180 HCC participants are needed to detect an absolute between-group difference of 15.5% (reducing the risk of LR from 20% in the control group to 4.5% in the treatment group). Assuming a 1:1 allocation ratio, we need 259 CRLM participants and 90 HCC participants in each group. This corresponds to a total of 518 CRLM and 180 HCC participants. Therefore, a total of 698 patients.

### Randomisation

Patients who meet the inclusion and exclusion criteria and sign the informed consent in the outpatient clinic are eligible for randomisation. Each patient will be assigned a code or identification number in strict sequential order. Randomisation will be conducted prior to surgery to allow for the preparation of specific devices. Patients will be allocated to either the AMC group or the control group at their respective centre by the study promoter, using an online computer-controlled permuted-block randomisation module (Research Electronic Data Capture, Vanderbilt University, Nashville, Tennessee, United States -RedCAp). This will be done in a 1:1 ratio without repositions, with block sizes varying between two and four patients. Randomisation will be stratified by centre and tumour type.

If a subject discontinues from the study, their subject number will not be reused, and re-entry into the study will not be permitted. However, during the recruitment period, a dropout subject may be replaced by a newly included patient who will receive the next available subject ID, in addition to the planned total number of subjects for that specific clinic.

### Blinding

It is not possible to double-blind this study due to the fact that it will be obvious for the study site team to which the patient has been allocated (ie patients in the test group will be treated with the new device in the operating room). However, patients will be unaware of the treatment allocation whenever possible. Inconsistency in this requirement will be reflected in the investigator file in the case report form (CRF).

### Data management, statistical analyses and quality assurance

#### Data management

All variables collected in the study will be recorded in the electronic CRF (eCRF), which will automatically transfer data to a database managed by the study coordinators, as outlined in the eCRF guidelines. Researchers and study monitors will have digital access to the eCRF and database to enter new patient data and review existing data during follow-up. Any additions or corrections made in the remote data entry system will be automatically logged in an audit trail.

A backup copy of the database will be created monthly. Both the eCRF and a copy of the prospective database will be retained for up to 10 years after the completion of the study and will be treated with the same confidentiality as other patient clinical data.

### Statistical methods

The main analyses will be conducted according to the intention-to-treat principle: all randomised patients will be included in the analyses based on the treatment allocation at randomisation. Multiple imputation will be used if more than 10% of patients have missing data on core variables, such as outcomes of interest and basic demographic characteristics.

Initially, the two groups will be compared for LR and other outcomes of interest using standard statistical techniques, without accounting for confounding variables (unadjusted analysis—eg, estimating HRs). OS, DFS, cancer-free survival (CFS) and HR will be compared between the two groups using two-sided unadjusted log-rank tests and Kaplan-Meier survival curves.

To adjust for confounding variables, we will analyse between-group differences in LR and other time-to-event outcomes (OS, DFS and CFS) using a mixed effects Cox proportional hazards model. This model will include a fixed effect for the intervention group and random effects for the site. We will adjust for the following variables: type of chemotherapy treatment, prior administration of adjuvant chemotherapy for primary cancer, previous chemotherapy for liver metastases, institution, type of resection, tumour stage, general coagulation method used and different RF application devices.

Effect estimates will be reported with 95% CIs and p values assuming a zero-effect hypothesis. All analyses will be performed using RStudio (Posit PBC, Boston, Massachusetts, United States)^29^ and relevant R packages.^30 31^ Trial results will be reported in accordance with the Consolidated Standards of Reporting Trials.^32^ We will explore the proportional hazards assumption using Schoenfeld residuals and, in case of violation, we will consider alternative models with time-varying coefficients for the explanatory variables that cause this violation and with restricted mean survival times.^33^

We are planning to conduct one interim analysis when half of the participants of the study (259 CLP and 90 HCC participants) have been recruited and finished a 6-month follow-up period using the O’Brien–Flemming stopping boundaries for efficacy.

### Quality assurance/data monitoring/data-sharing plan

All patient data related to the study will be recorded on eCRFs. The investigator is responsible for ensuring that data entries are accurate and correct, either by physically or electronically signing the eCRF. Guidance on completing eCRFs will be provided in the Trial Master File.

The investigator must allow for study-related monitoring, audits, Research Ethics Committee and National Competent Authority (REC/NCA) reviews and regulatory agency inspections and provide direct access to source data documents. The monitoring strategy, including definitions of study-critical data items and processes, risk management and mitigation strategies, and monitoring techniques (central, remote or on-site), is detailed in the monitoring plan. The sponsor or their designee is responsible for data management, including quality checking of the data. The sponsor retains accountability for actions delegated to other individuals, such as contract research organisations.

Records and documents, including signed ICFs, must be retained by the investigator for 10 years after the completion of the study, unless local regulations or institutional policies mandate a longer retention period. Records may not be destroyed during this retention period without written approval from the sponsor. Additionally, records may not be transferred to another location or party without written notification to the sponsor.

The data generated by this project, along with supporting information, will be deposited in the CORA Research Data Repository (https://dataverse.csuc.cat/), a multidisciplinary repository supporting FAIR (Findable, Accessible, Interoperable and Reusable) principles and EOSC (European Open Science Cloud) guidelines. This repository ensures long-term, open access to the data with persistent identifiers (digital object identifier (DOIs)) for easy citation and reuse. Metadata standards such as the Data Documentation Initiative (DDI), Dublin Core and DataCite are used to enhance data discoverability and interoperability.

Data will be preserved and made publicly accessible for at least 10 years under CC0 (Creative Commons Zero) or other appropriate licences. Due to the small scale of the study, no data access committee is required. This approach aligns with the European FAIR principles to promote data findability, accessibility, interoperability and reusability.

### Serious adverse events

A serious adverse event (SAE) is an adverse effect that meets one or more of the following criteria: (1) it leads to the patient’s death, (2) there is an imminent risk of death, (3) it requires hospitalisation or prolongation of hospitalisation, (4) it involves a disability or significant persistent sequela, and (5) it constitutes a major medical event that is life-threatening or may require medical intervention to prevent any of the above outcomes.

Any SAE must be recorded on the eCRF of the patient, including the start time, action taken and an assessment of whether it qualifies as an SAE. The SAE committee will review these events, and the trial will proceed if more than 10% of the patients treated in the initial phase experience SAEs.

In our previous evaluation of the method, we did not observe any significant adverse events (AEs) or adverse device effects that could be attributed to the device or its method of use.^34^ Nonetheless, we provide a list of serious surgery-related complications that could potentially be associated with the procedure, and which may also encompass possible device-related AEs (Boxes1  1).

Box 1 **Potential serious adverse events**

- Postoperative bleeding with replacement >4 units of erythrocyte concentrates, severe sepsis (according to the American College of Chest Physicians/Society of Critical Care Medicine). However, evaluating this variable, we must bear in mind that with conventional methods of liver transection, 16% of our patients must be transfused.
- Subphrenic or perihepatic abscess requiring drainage.
- Relaparotomy connected with the procedure.
- A biliary fistula for more than 10 days with discharge >10 mL/day.
- Transient liver failure (bilirubin >10 mg/dL lasting >3 days).
- Renal failure requiring dialysis.
- Respiratory failure with renewed necessary mechanical ventilation.
- Death of the patient as a result of the operation.

### Duration and schedule

The overall trial is anticipated to take 5 years to complete, including the preparation of manuscripts and data analysis. Recruitment for the trial began in November 2023 at the Hospital del Mar in Barcelona. The recruitment period will be 30 months followed by 3 years of follow-up. The recruitment period is projected to continue until June 2026. The follow-up period will be completed by June 2029. Data analysis and manuscript preparation are expected to be completed within 3 months following the end of the follow-up period.

### Ethics and dissemination

The study sponsor is responsible for obtaining approval from the Institutional Ethics Committees (IECs) in each participating country. The protocol has already been approved by the Institutional Review Board at the sponsor site Hospital del Mar, Barcelona – Spain (Comité de Ética de la Investigación con Medicamentos CEIm_PSMAR2023/11115), as well as by the IECs in all participating countries, including Italy (Comitato Etico Territoriale Lazio Area 2–157.24 PUD CET2), France (Comité de Protection des Personnes Île-de-France II – 2024-A00906-41), Poland (Komisja Bioetyczna przy Warszawskim Uniwersytecie Medycznym – KB118/2024), Slovenia (Komisija Republike Slovenije za medicinsko etiko – 0120-59/2024-2711-7), Switzerland (Kantonale Ethikkommission – BASEC-Nr 2024-D0088), and Greece (Ethics Committee of the University Hospital ‘Laiko’ – 279; Ethics Committee of the University General Hospital of Larissa – 26203; Ethics Committee of the Anti-Cancer Hospital of Piraeus ‘Metaxa’ – 12361/27-6-25).

Although the surgical procedure itself is not modified by the clinical trial, the usual informed consent process will be used at each centre. Once the standard surgical consent is signed, patients will be asked to provide specific consent for participation in the study, with information about the possibility of being assigned to either study group. The principal investigator is responsible for informing the IEC of any protocol amendments in accordance with local requirements. Civil liability insurance will be in place for the study.

This project will adhere to national and international guidelines, including the Declaration of Helsinki (64th General Assembly, Fortaleza, Brazil, October 2013) and the regulations governing studies with biological samples, as outlined in Law 14/2007 on Biomedical Research. All amendments to the research protocol will be notified to the IEC that provided the favourable opinion. Non-substantial amendments will be recorded and filed by the sponsor but will not be notified to the IEC.

Data confidentiality is ensured in compliance with current regulations, including Organic Law 3/2018 of 5 December, on the Protection of Personal Data and Guarantee of Digital Rights, and Regulation (EU) 2016/679 of the European Parliament and Council of 27 April 2016, on Data Protection. Patients will be assigned a unique identifier by the sponsor. Records and datasets transferred to the sponsor will contain only this identifier; patient names or any other identifying information will not be included. Sponsor staff who access personal data will be required to maintain confidentiality. Data collected will be limited to what is necessary to achieve the study objectives. Patients will be informed that their personal study-related data will be used by the sponsor in accordance with local data protection laws. The level of disclosure will be explained to patients, and they will be required to consent to the use of their data as described in the informed consent document.

### Dissemination and publication policy

The dissemination strategy aims to engage stakeholders and facilitate the transfer of knowledge to support the use of study findings. The primary audiences for dissemination will include health organisations, the medical research community, the medical device industry and social stakeholders such as policymakers and key opinion leaders. A comprehensive dissemination strategy will be developed to maximise the reach and impact of the clinical results.

All information about LIVERATION can be assessed at the project’s website https://liveration.eu/team/

### Patient and public involvement

Patients were not directly involved in the design and conduct of this research. However, patients will be asked to support the setting of the outcome measures for the QoL questionnaires and help to decide about the most appropriate ones. Once the trial has been published, results will be communicated to keep people informed throughout the project, reporting negative and positive results.

## Supplementary material

10.1136/bmjopen-2025-100518 online supplemental file 1

10.1136/bmjopen-2025-100518 online supplemental file 2

10.1136/bmjopen-2025-100518 online supplemental video 1
