# Supplementary material for: LIVERATION trial: a multicentre European randomised study on radiofrequency margin coagulation and its impact on oncological outcomes after liver surgery – study protocol
Source: BMJ Open. 2025 Nov 24;15(11):e100518. doi: 10.1136/bmjopen-2025-100518 (PMC12645656; doi:10.1136/bmjopen-2025-100518)
Supplement: online supplemental file 2 [file bmjopen-15-11-s002.pdf]

# PATIENT INFORMATION AND INFORMED CONSENT FORM

| | |
|---|---|
| **TITLE OF THE STUDY** | Unveiling the impact of radiofrequency on the liver surgery: the key to reducing local recurrence? |
| **STUDY CODE** | LIVERATION |
| **Clinicaltrials.gov Identifier** | NCT05492136 |
| **SPONSOR** | Consorci Mar Parc de Salut de Barcelona |
| **PRINCIPAL INVESTIGATOR:** | |
| **SITE:** | |

## Introduction

We are writing to inform you about a research study in which you are invited to participate. The study has been approved by the Ethics Committee for Research involving medicinal products of the Hospital del Mar in accordance with the current legislation of Royal Decree 192/2023 of 21 March, which regulates medical devices.

Our intention is that you receive sufficient and correct information so that you can decide whether or not to take part in this study. To this end, please read this information sheet carefully and we will answer any questions you may have. In addition, you can consult with the people you consider appropriate.

## Voluntary participation

You should be aware that your participation in this study is voluntary and that you may decide NOT to participate or to change your decision and revoke your consent at any time, without altering your relationship with your doctor or causing any harm to your health care.

## Objective

Liver cancer is the sixth most commonly diagnosed cancer and the third leading cause of cancer deaths in the world as reported in 2020. Hepatocellular carcinoma is the main subtype of primary liver carcinoma and accounts for approximately 85% of cases. In addition, the liver is also the main site of metastasis for colorectal cancer, the third most frequent malignancy worldwide.

Liver resection or hepatectomy, a surgical intervention to remove the tumour-bearing part of the liver, remains the standard treatment for hepatocellular carcinomas and metastatic liver tumours of colorectal origin. However, even after surgeries, hepatocellular carcinoma and colorectal liver metastases still have a high recurrence rate. Further research is needed to find a solution to this problem. Based on research with hyperthermic therapies, we believe that, after surgery, additional coagulation of the remaining liver margin with a radiofrequency device could decrease the rate of tumour recurrence. Additional radiofrequency margin coagulation involves the application of high-frequency electromagnetic waves to the liver tissue at the margins of the tumour. The mechanism of action of this device is to produce a thermal coagulation of the tissue that causes tissue cell death, thus causing the death of any cancer cells that may have remained in the area close to the resected tumour. We have conducted an experimental study in which we have observed a reduction in tumour recurrence with the study methodology, but to further strengthen this hypothesis and determine its impact on the patient's quality of life, a multicentre clinical trial is needed to compare the study methodology with conventional methods.

Therefore, the aim of this study is to determine whether the technique called additional margin coagulation using a radiofrequency device decreases the local recurrence of this type of tumour.

## Description of the study

You are invited to participate in this study because you are of legal age and have been diagnosed with liver metastases, cancer of colon or rectum origin, or cancer that has arisen directly in the liver (also called hepatocarcinoma) without prior treatment with local therapy or liver resection.

This study will be conducted in 7 European countries and will include a total of 698 patients. After agreeing to participate in the study by signing this patient information sheet, you will be randomly assigned, as well as when a coin is flipped, to one of the two treatment groups in the study:

- Hepatectomy with conventional methods according to the surgeon's preference **without** margin clotting after surgery
- Hepatectomy with conventional methods according to the surgeon's preference, **and** additional margin coagulation with a radiofrequency-assisted device

You will have an equal chance of being assigned to one group or the other. You will not know which treatment group you have been assigned to until the end of the study. Only the study doctors treating you will know this information.

## Study activities

The study has a duration of approximately 3 years per patient and a minimum of 7 visits during that period.

At the first study visit, you will be asked to participate in the study. If you decide to participate, you will be given all the information about the study and you will sign the informed consent form attached to this information sheet. During this visit you will also undergo an imaging test (CT scan) or magnetic resonance imaging (MRI) to study the type of tumour in depth. Your medical history will be reviewed, a physical examination will be carried out, including an electrocardiogram and a blood draw to check that you meet the necessary criteria to be included in the study. If so, you will be assigned to a treatment group. To assess the impact of the treatment on your quality of life and to take your perspective into account in decision-making during the clinical trial, you will also be asked (with your consent) to answer two questionnaires during the first visit and at some of the follow-up visits after surgery (1 month, 1 year, 2 years and 3 years after surgery). You will be able to complete these questionnaires either electronically or on paper. If you choose to complete them electronically, you will need to provide your email address so we can send you a link to the platform where the questionnaires are hosted. Your contact information will be stored securely on this platform, and only the research team will have access to it. If you agree to use the platform, you will also receive a user guide to help you.

As part of the study, you will be asked to complete two specific questionnaires: the Client Service Receipt Inventory (CSRI) and the EQ-5D-5L. These questionnaires are used to understand the economic and quality-of-life impact of the treatment being studied. You will be asked to complete them during your first visit (before surgery), and again during follow-up visits at 1 month, 1 year, 2 years, and 3 years after surgery. The information collected will help us understand the relationship between the costs of the treatment and its impact on quality of life and health. This is very important to evaluate how effective and efficient the treatment is from a public health perspective.

The next visit of the study will correspond to the surgical intervention visit in which you will be operated according to the treatment group to which you have been assigned. A blood test will also be taken at this visit. The surgical samples will continue their course in the Pathological Anatomy Service, as well as for the biobank, as will the rest of the samples and tissues sent for study. The surplus sample that is not used in the study will be destroyed when the analysis of the results of the study has been completed.

Follow-up after discharge to evaluate the clinical and morphological evolution of the liver by means of imaging (CT) or magnetic resonance imaging (MRI) will be performed at 30 and 90 days after surgery, at one year, at two years and at three years, which does not differ from the usual practice after this type of surgery. A blood draw will also be performed at these visits. In the visits made at 30 and 90 days, in addition, a blood sample will be taken again for the analysis of the presence of tumor DNA for research related to the study of your disease and treatment. During your 30-day post-surgery follow-up visit, you will also be asked to answer a questionnaire about your experience with the care you received. If, due to some extraordinary circumstance,

you are unable to fill out the questionnaires in person, or you have any questions about them, you may be called to complete them by telephone.

## Risks and discomforts arising from participation in the study

The radiofrequency device by means of which the intervention will be performed is currently authorised and commercially available.

The additional coagulation technique to be used in the study group has already been tested in other studies and does not entail additional risks compared to the traditional technique where this additional coagulation of the tumour margin to be removed is not performed.

The complications you may experience are those of hepatectomy (cutting and removal of a small part of the liver) and the possibility of complications is similar whether you are assigned to the group where the conventional technique is used or to the group where the new technique under study is being tested.

Potential complications of hepatectomy include postoperative haemorrhage, transient liver failure, hepatic duct injury, biliary fistula, abdominal sepsis, wound infection and spread of infection in the body, renal failure requiring dialysis, and death as a consequence of the operation.

The risks and discomforts of tests such as blood draws can include dizziness, fainting, lightheadedness, bruising or infection at the site of infection.

<u>Pregnancy warning:</u>

If pregnancy occurs during your participation in the study, you should inform your doctor immediately in order to receive appropriate medical care.

## Potential benefits

You may not directly benefit from your participation in this study. However, the identification of a methodology that decreases tumour recurrence may benefit other patients in the future.

In a previous study, the technique of additional margin coagulation in liver resections was shown to decrease local recurrence.

Moreover, their participation in this study does not add any additional risk or cost to routine clinical practice.

## Contact in case of questions

If you have any questions or need more information during your participation, please contact the principal investigator of the study, ____________________________________, from __________________________by writing to ________________ or calling the ___________.

## Alternative treatments

If you decide not to take part in this research, you will continue to receive your usual treatment for the disease you have.

## Expenses and financial compensation

The clinical trial is funded by the European Commission and the study is sponsored and conducted by the Hospital del Mar Medical Research Institute.

For the study to be carried out, the study sponsor has signed a contract with the study doctor and the centre where the study is to be carried out. You will not have to pay for treatments or specific tests for the study.

You will not receive any financial compensation for your participation in the study.

## Protection of personal data

Both the promoter and the centre shall ensure that the principles of data protection legislation, both national and European, are complied with:

- Regulation (EU) 2016/679 of the European Parliament and of the Council of 27 April 2016 (GDPR) on the protection of individuals with regard to the processing of personal data and on the free movement of such data.
- Organic Law 3/2018 of 5 December 2018 on the Protection of Personal Data and Guarantee of Digital Rights (LOPDPGDD) and any other implementing regulations.

It is therefore important that you are aware of the following information:

In accordance with current European and national regulations on Personal Data Protection, the personal data obtained will only be those necessary for the purposes of the study. In addition to the rights you already know (access, rectification, opposition and cancellation of data) you will now also be able to limit the processing of data that are incorrect, request a copy or transfer them to a third party (portability). You will also have the right to withdraw your consent to the processing of the data, although such withdrawal may result in the termination of your participation in the trial. To exercise your rights, please contact the researcher.

We remind you that the data cannot be deleted, even if you stop participating in the trial or, even if you withdraw your consent to data processing, in order to ensure the validity of the research and to comply with legal obligations and drug authorisation requirements. You also have the right to contact the Data Protection Agency if you are not satisfied. Both the Centre and the Sponsor are respectively responsible for the processing of your data and undertake to comply with the applicable data protection regulations. The data collected for the study will be identified by a code, so that it does not include information that can identify you, and only the principal investigator(s)/collaborating investigator(s) will be able to relate this data to you and your medical history. Therefore, your identity will not be disclosed to anyone else, except to health authorities, when necessary or in cases of medical emergency. Research Ethics Committees, representatives of the inspecting Health Authority and personnel authorised by the Sponsor may only verify personal data, clinical trial procedures and compliance with standards of good clinical practice (while maintaining confidentiality of information).

The investigator and the sponsor are obliged to keep the data collected for the study for at least 10 years after the end of the study. Thereafter, your personal data will only be retained by the centre for your health care.

If we transfer your encrypted data outside the EU to our group entities, service providers or scientific researchers who collaborate with us, the participant's data will be protected with safeguards such as contracts or other mechanisms by data protection authorities. In any case, the recipients of the data will not have access to the code that allows their data to be linked to you; Only your doctor or hospital staff have access to this key. If the data is transferred to a country where the legislation is not as strict as that in Spain, the sponsor will either sign a data sharing agreement with the receiving research partner that incorporates standard contractual clauses approved by Spain, taking into account that its legislation integrates all the clauses of the

European GDPR, or use another transfer mechanism that safeguards the way in which your personal data is processed.

If you would like to know more in this regard, please contact the Data Protection Officer of the developer (protecciodedades@imim.es). If you would like to know more about the platform used to complete the questionnaires electronically, you can contact the Data Protection Officer of FAD (protecciondatos@fadq.org). In accordance with current European and national regulations on Personal Data Protection, the personal data collected will be those necessary for the purposes of the study. In accordance with current legislation, you have the right to be informed of any health-relevant data collected in the course of the study. This information will be communicated to you if you so wish; in the event that you prefer not to be informed, your decision will be respected.

## More information on this study

A description of this clinical trial will be published on clinicaltrials.gov This website will not contain any identifying information. A summary of the results will be published on this registry at the end of the study. You will be able to consult this website at any time.

# INFORMED CONSENT

| Title of the study | Unveiling the impact of radiofrequency in surgery liver: the key to reducing local recurrence? |
|---|---|
| **Clinicaltrials.gov Identifier** | NCT05492136 |
| **Protocol code** | LIVERATION |

I, ________________________________ (name and surname)

- I have read the information sheet provided to me
- I have been able to ask questions about the study
- I have received sufficient information about the study
- I have spoken to: _______________________________________ (name of researcher).

**I understand that:**

1. My participation is voluntary.
2. That it is of no direct benefit to me.
3. That refusal to participate in this study will not affect my medical care.
4. That the information obtained from this study is confidential.
5. That the surplus sample that will not be used in the study will be destroyed when the results of the study are finalised.
6. That the procedure under study does not involve any additional risks to those of standard clinical practice.
7. That participation in the study does not involve additional costs beyond those of normal clinical practice.
8. I consent to an additional blood sample being taken during study visits 1, 3, and 4. This sample will be stored and used in future research related to the detection of circulating tumor DNA.

   Yes ☐ No ☐

9. I agree that, in exceptional situations, I may be contacted by telephone (please indicate yes or no and the contact telephone number) to carry out or clarify questions regarding the experience and quality of life questionnaires.

   **Yes ☐ No ☐ Contact telephone number: ______________________________________**

10. I consent to participate in completing the study questionnaires through an electronic platform, for which I provide my email address (please indicate Yes or No and provide your contact email address).

    **Yes ☐ No ☐ Contact email: _________________________________________________**

**I authorise my inclusion in this study**

Date: ________________

| | |
|---|---|
| ___________________________________ | ____________________________________ |
| Name of participant | Signature of participant |

Date: ________________

| | |
|---|---|
| _____________________________________ | ____________________________________ |
| Name of researcher | Signature of researcher |
| _____________________________________ | ___________________________________ |
| Signature of the legal representative witness (if necessary) | Relation of representative with the participant |
