# Supplementary material for: LIVERATION trial: a multicentre European randomised study on radiofrequency margin coagulation and its impact on oncological outcomes after liver surgery – study protocol
Source: BMJ Open. 2025 Nov 24;15(11):e100518. doi: 10.1136/bmjopen-2025-100518 (PMC12645656; doi:10.1136/bmjopen-2025-100518)
Supplement: Supplementary file 4 [file bmjopen-2025-100518.reviewer_comments.pdf]

PEER REVIEW HISTORY

BMJ Open publishes all reviews undertaken for accepted manuscripts. Reviewers are asked to complete a checklist review form and are provided with free text boxes to elaborate on their assessment. These free text comments are reproduced below.

ARTICLE DETAILS

Title (Provisional)

"LIVERATION Trial: A Multicenter European Randomized Study on Radiofrequency Margin Coagulation and its Impact on Oncological Outcomes After Liver Surgery – Study Protocol"

Authors

Luque Villalobos, Eduardo; Ielpo, Benedetto; Aldrighetti, Luca; Anselmo, Alessandro; Beghdadi, Nassiba; Berardi, Giammauro; Briceño, Francisco; Ciria, Ruben; Dorcaratto, Dimitri; Durczynski, Adam; Ettorre, Giuseppe Maria; Delvecchio, Antonella; Ferri, Valentina; Grąt, Michał; Garces, Marina; Grochola, Lukasz Filip; Hogendorf, Piotr; Izzo, Francesco; Kobryn, Konrad; Kontis, Elissaios; Korkolis, Dimitris; Król, Robert; Lesurtel, Mickael; Lopez-Ben, Santiago; Christogiannis, Christos; Koutsiouroumpa, Ourania; Mavridis, Dimitris; Memeo, Riccardo; Murawa, Dawid; Machairas, Nikolaos; Mora, Isabel; Muñoz, Elena; Orrego, Carola; Dantas, Carina; Fuste, Carlos; García Picazo, Alberto; Pessaux, Patrick; Pilat, Tomasz; Pittau, Gabriella; Radosevic, Aleksandar; Ratti, Francesca; Sotiropoulos, Georgios; Toutouzas, Konstantinos; Trotovsek, Blaz; Wystrychowski, Wojciech; Zacharoulis, Dimitris; Vicente, Emilio; Burdio, Fernando; Sánchez Velázquez , Patricia

---

VERSION 1 - REVIEW

**Reviewer**	**1**
**Name**	**Dakua, Sarada Prasad**
**Affiliation**	**Hamad Medical Corporation**
**Date**	**16-Jul-2025**
**COI**	**None**

The paper has addressed a nice issue; however, I have the below comments, which the authors should rebut:

1- Liver resections for colorectal liver metastases and HCC are specialized procedures often concentrated in high-volume centres, which may limit the recruitment pace.

2- Strict eligibility rules may further restrict the pool of eligible participants.

3- Variability in resection methods, use of ablation, or minimally invasive surgery across centres can affect outcomes.

4- Inconsistent follow-up imaging quality or pathological assessment could skew local recurrence data.

5- Underreporting or misclassification due to lack of centralized adjudication.

6- Qality of life is a bit subjective.

7- The investigators must ensure all centres to use consistent definitions, CRFs (case report forms), and datebases.

8- There could be missing data, at least high potential due to long-term follow-up, patient dropouts, or death.

Reviewer 2

Name Li, Jianwei

Affiliation Chinese Research Group for Minimally Invasive Anatomical Liver Resection

Date 04-Aug-2025

COI None

This study (the LIVERATION trial) focuses on the impact of radiofrequency edge ablation on oncological outcomes after liver surgery. It is designed as a multicenter randomized controlled trial, with clinical value, a reasonable methodological framework, and funded by the Horizon Europe program, demonstrating high innovation and feasibility. However, the seven comments mentioned above pertain to the refinement of study details (such as standardized procedures, the rationality of sample size, and bias control), which the authors need to supplement and modify to enhance the study's rigor and the reliability of the results. After review and confirmation of the corrections, publication may be considered.

1. Clarify the size and location range of included tumors: The inclusion criteria in the text mention colorectal liver metastases (CRLM) and hepatocellular carcinoma (HCC), but do not specify the exact range of tumor size and location (such as proximity to major vessels or bile ducts). Since tumor size and location can significantly affect the feasibility and efficacy of radiofrequency edge ablation (for instance, the risk of thermal injury increases when close to major vessels), it is recommended to specify the diameter range of included tumors (such as ≤5cm) and the safety distance from important structures to improve the homogeneity of the study.

2. Refine the standardized procedures for operating radiofrequency devices: Although the parameters of Coolingbis® and Aquamantys® (such as power and operation time) were described, the training and certification of the operating physicians in the multi-center study,

the frequency of equipment calibration, and the quality control measures for operational consistency were not mentioned. It is recommended to supplement the "standardized operation plan", including unified training records, equipment calibration logs, and regular cross-center operational consistency checks to reduce bias caused by operational differences.

3. Standardize the imaging assessment criteria for local recurrence (LR): The article defines LR as "growth or enhancement of the tumor at the resection margin", but does not specify the specific assessment parameters for CT/MRI (such as slice thickness, enhancement phase, and qualifications of the imaging interpreter). Differences in image interpretation among multiple centers may affect the consistency of LR determination. It is recommended to develop a unified imaging assessment manual, clearly defining scanning parameters, interpretation procedures, and a third-party blind review mechanism.

4. Provide a reasonable explanation for the relatively small sample size of HCC: Among the total 720 cases, HCC only accounts for 192 cases (approximately 26.7%), significantly fewer than CRLM (528 cases). HCC has high heterogeneity (such as coexisting liver cirrhosis, satellite nodules, etc.), and a smaller sample size may reduce the statistical power of subgroup analysis. It is suggested to provide the basis for sample size allocation (such as based on the difference in LR occurrence rates in the past), and explain whether stratified analysis or an expanded HCC enrollment plan will be adopted to compensate for the potential lack of power.

5. Strengthening bias control measures in single-blind design: As surgeons are aware of the group allocation (the intervention group uses radiofrequency devices), it may affect other operations during the surgery (such as the extent of liver resection and hemostasis methods) except for marginal coagulation. It is recommended to supplement a "standardized surgical protocol", clearly stating that all steps except marginal coagulation (such as hepatic portal occlusion and hemostasis device selection) must strictly follow the center's routine, and confirm the consistency of the process through random checks of surgical videos to reduce subjective bias.

6. Adjust the time points for quality of life (QoL) assessment: The article mentions that QoL assessment excludes the day of surgery and 90 days post-surgery. However, 90 days post-surgery is a period of high risk for liver function recovery and complications, which significantly affects patients' QoL. Omitting this time point may lead to incomplete data. It is recommended to include the QoL assessment at 90 days post-surgery, or clearly state the reasons for exclusion (such as low patient follow-up compliance) in the methods section, and supplement a sensitivity analysis plan.

7. Refinement of monitoring for radiofrequency-related adverse events: Although serious adverse events (SAEs) were defined in the safety assessment, no specific list of adverse events unique to radiofrequency treatment (such as bile fistula, bleeding, and thermal injury to adjacent organs caused by excessive coagulation of marginal tissues) was provided. It is

recommended to supplement a "list of radiofrequency-related adverse events", including symptoms, diagnostic criteria, and grading, to ensure the specificity and completeness of safety data.

VERSION 1 - AUTHOR RESPONSE

Reviewer #1:

1- Liver resections for colorectal liver metastases and HCC are specialized procedures often concentrated in high-volume centres, which may limit the recruitment pace. **Thank you for this observation. We have addressed this point in the *Study Design* subsection of the *Materials and Methods* section, where we acknowledge the potential impact of this factor on the recruitment timeline.**

2- Strict eligibility rules may further restrict the pool of eligible participants. **Thank you for this important observation. In the case of the LIVERATION study, we have opted for a pragmatic design with intentionally broad inclusion criteria, aiming to ensure that the findings are generalizable to routine clinical practice. We acknowledge that this approach may introduce certain methodological challenges, such as potential heterogeneity and confounding. However, we have incorporated specific strategies within statistical analysis to address and minimize these risks.**

3- Variability in resection methods, use of ablation, or minimally invasive surgery across centres can affect outcomes. **Thank you for this valuable and insightful observation. We agree that variability in resection methods, the use of ablation, and minimally invasive surgery across centers can influence outcomes. Following the initial analysis, we plan to conduct a secondary analysis adjusted for potential confounding variables, including: type of chemotherapy regimen, prior administration of adjuvant chemotherapy for the primary tumor, previous chemotherapy for liver metastases, participating institution, type of liver resection, tumor stage, general coagulation method used, and the type of radiofrequency ablation device. In addition, we will strongly encourage centers to avoid the use of radiofrequency instruments for hepatic transection. This approach aims to minimize heterogeneity and enhance the robustness and validity of our findings.**

4- Inconsistent follow-up imaging quality or pathological assessment could skew local recurrence data. **Thank you for your comment. As previously mentioned, all participating centres are high-volume liver surgery centres, each performing more than 50 liver resections per year and therefore with recognized expertise. These centres also have radiology and pathology departments with extensive experience in hepatic oncology. Radiology assessment of the transection area on the 30 days postoperative CT will be assessed in the promotor centre. To ensure the consistency and quality of follow-up imaging and pathological assessments, regular monitoring visits will be conducted at all sites to verify proper equipment calibration and protocol adherence. This is mentioned in the Quality assurance/Data monitoring sub-section.**

5- Underreporting or misclassification due to lack of centralized adjudication. **Thank you for raising this important point. As previously noted, we acknowledge the possible impact of conducting patient follow up across multiple centers. To mitigate this risk, we have established clearly defined and objective primary and secondary endpoints, as outlined in detail in the study protocol Furthermore, to enhance data validity and ensure consistency across sites, external monitoring and auditing will be carried out by a Contract Research Organization (CRO) at every participating center.**

These measures are described in the *Data Capture and Trial Endpoints* and in the Quality assurance/Data monitoring subsections.

We believe this approach will minimize variability and contribute to the reliability of the reported outcomes.

6- Qality of life is a bit subjective. **Thank you for highlighting this point. While we acknowledge that quality of life is inherently subjective, we believe it is a key outcome to assess in patients undergoing liver resections, which are often complex procedures. To ensure a reliable and standardized evaluation, we are using the QLQ-C30 and QLQ-HCC18 questionnaires, validated tools widely used in oncology research, including in multicultural settings and specifically in patients with liver tumors, as it can be noted in the data capture and trial endpoints section.**

7- The investigators must ensure all centres to use consistent definitions, CRFs (case report forms), and datebases. **Thank you for your comment. Clear definitions for both primary and secondary endpoints are specified in the study protocol. A single, standardized case report form (CRF) will be used across all participating centers to ensure uniform and well-structured data collection. Each center's principal investigator, as well as the monitoring agencies, will have access to the CRF to verify the accuracy and completeness of the collected data. These procedures are detailed in the section titled *"Data Management, Statistical Analyses, and Quality Assurance."***

8- There could be missing data, at least high potential due to long-term follow-up, patient dropouts, or death. **We appreciate this important observation. We have revised the sample size calculation accordingly, as reflected in the latest version of the protocol (version 1.4, dated May 28, 2025). Taking into account an anticipated attrition rate of up to 10%, due to medium- to long-term follow-up, patient dropouts, and mortality, we have adjusted the sample size to 518 patients with colorectal liver metastases and 180 patients with hepatocellular carcinoma. These numbers are calculated to ensure 5% statistical significance and to detect a risk reduction of 6.9% and 15.5%, respectively. These updates are detailed in the section *"Calculation and Justification of the Sample Size."* In addition, we have planned retention strategies to minimize patient loss during follow-up.**

***Reviewer #2*:**

1. Clarify the size and location range of included tumors: The inclusion criteria in the text mention colorectal liver metastases (CRLM) and hepatocellular carcinoma (HCC), but do not specify the exact range of tumor size and location (such as proximity to major vessels or bile ducts). Since tumor size and location can significantly affect the feasibility and efficacy of radiofrequency edge ablation (for instance, the risk of thermal injury increases when close to major vessels), it is recommended to specify the diameter range of included tumors (such as ≤5cm) and the safety distance from important structures to improve the homogeneity of the study. **Thank you for this insightful comment. As stated in the inclusion criteria, all patients with resectable colorectal liver metastases (CRLM) or hepatocellular carcinoma (HCC) are eligible for inclusion, regardless of tumor size or location. While we acknowledge that differences in tumor size and proximity to critical structures (such as major vessels or bile ducts) may introduce some heterogeneity, this reflects the real world setting in which these procedures are performed. To address this variability, we have planned adjusted analyses that will account for potential confounders, including tumor size, anatomical location, and distance to major vascular or biliary structures. This approach aims both to minimize heterogeneity bias and to assess the true clinical utility of radiofrequency assisted liver resection in everyday surgical practice.**

2. Refine the standardized procedures for operating radiofrequency devices: Although the parameters of Coolingbis® and Aquamantys® (such as power and operation time) were described, the training and certification of the operating physicians in the multi-center study, the frequency of equipment calibration, and the quality control measures for operational consistency were not mentioned. It is recommended to supplement the "standardized operation plan", including unified training records, equipment calibration logs, and regular cross-center operational consistency checks to reduce bias caused by operational differences. **Thank you for this important observation. We have clarified this point in the section *Trial-Specific Interventions*. If additional details regarding the training and monitoring visits are required, please let us know, and we will be happy to provide them.**

3. Standardize the imaging assessment criteria for local recurrence (LR): The article defines LR as "growth or enhancement of the tumor at the resection margin", but does not specify the specific assessment parameters for CT/MRI***sasa (such as slice thickness, enhancement phase, and qualifications of the imaging interpreter). Differences in image interpretation among multiple centers may affect the consistency of LR determination. It is recommended to develop a unified imaging assessment manual, clearly defining scanning parameters, interpretation procedures, and a third-party blind review mechanism. **Thank you for this valuable observation. We have incorporated the requested specifications regarding image acquisition and evaluation for CT and MRI into the *Primary Outcomes* section of the manuscript. These additions include details on slice thickness, contrast phases, and the qualifications of the image interpreters, as well as the inclusion of a centralized, blinded review by an experienced radiologist to ensure consistency across participating centers. If further clarification is required, we would be happy to provide additional information.**

4. Provide a reasonable explanation for the relatively small sample size of HCC: Among the total 720 cases, HCC only accounts for 192 cases (approximately 26.7%), significantly fewer than CRLM (528 cases). HCC has high heterogeneity (such as coexisting liver cirrhosis, satellite nodules, etc.), and a smaller sample size may reduce the statistical power of subgroup analysis. It is suggested to provide the basis for sample size allocation (such as based on the difference in LR occurrence rates in the past), and explain whether stratified analysis or an expanded HCC enrollment plan will be adopted to compensate for the potential lack of power. **Thank you for this valuable comment. In the latest revision of the protocol, we have updated the sample size calculation. The total sample now consists of 698 patients: 518 with colorectal liver metastases (CRLM) and 180 with hepatocellular carcinoma (HCC).**

The smaller sample size for the HCC group is based on two key factors. First, the incidence of HCC eligible for surgical resection is considerably lower than that of resectable CRLM, as outlined in the referenced literature. Second, although HCC is less frequent, it presents a higher rate of local recurrence, primarily due to the presence of microscopic satellite nodules at the periphery of the lesion. This combination of lower incidence but higher recurrence risk leads to higher clinically relevant difference and therefore to a smaller sample size, while still ensuring sufficient statistical power to detect clinically meaningful differences.

Furthermore, both cohorts will be analyzed separately. Each will undergo an initial unadjusted analysis followed by an adjusted analysis accounting for relevant confounders. In the HCC group, these confounding variables include the presence of cirrhosis, portal hypertension, and satellite nodules. This approach aims to maximize the validity and interpretability of subgroup findings despite the sample size differences.

5. Strengthening bias control measures in single-blind design: As surgeons are aware of the group allocation (the intervention group uses radiofrequency devices), it may affect other operations during the surgery (such as the extent of liver resection and hemostasis methods) except for marginal coagulation. It is recommended to supplement a "standardized surgical protocol", clearly stating that all steps except marginal coagulation (such as hepatic portal occlusion and hemostasis device selection) must strictly follow the center's routine, and confirm the consistency of the process through random checks of surgical videos to reduce subjective bias. **Thank you for this valuable observation. As correctly noted, due to the single-blind design of the study, the surgical team will be aware of the patient's group allocation. However, since this is a pragmatic trial, all surgeries will be performed according to each center's routine clinical practice, regardless of group assignment, with the primary objective of achieving an adequate oncological resection in all cases.**

We fully agree on the importance of minimizing potential bias. To standardize the technique of additional margin coagulation, we have implemented the following measures:

- **Prior to the initiation of patient recruitment at each participating center, a training and certification visit will be conducted by the study sponsor (IMIM) on the appropriate use of the radiofrequency device.**
- **Supporting video materials demonstrating proper device use will be provided to all centers.**
- **Centers will be encouraged not to use radiofrequency devices for liver transection, in order to limit their use specifically to marginal coagulation as per protocol.**

These steps aim to ensure procedural consistency and reduce the risk of bias introduced by variations in surgical technique.

These additions have been included in the section *"Trial-Specific Interventions"* for the radiofrequency group. We will consider the implementation of random surgical video assessments to verify adherence to standard procedures.

6. Adjust the time points for quality of life (QoL) assessment: The article mentions that QoL assessment excludes the day of surgery and 90 days post-surgery. However, 90 days post-surgery is a period of high risk for liver function recovery and complications, which significantly affects patients' QoL. Omitting this time point may lead to incomplete data. It is recommended to include the QoL assessment at 90 days post-surgery, or clearly state the reasons for exclusion (such as low patient follow-up compliance) in the methods section, and supplement a sensitivity analysis plan. **Thank you for this thoughtful observation. This issue was indeed considered during the initial design of the protocol. However, we determined that the most significant postoperative complications, those likely to substantially affect quality of life, typically occur within the first 30 days after surgery. Therefore, we believe that the QoL assessment scheduled at 30 days postoperatively will adequately capture the major early impacts on patients' quality of life.**

Nonetheless, we acknowledge that the 90-day period may also reflect ongoing recovery dynamics, and we will take this into consideration in future amendments to the protocol.

7. Refinement of monitoring for radiofrequency-related adverse events: Although serious adverse events (SAEs) were defined in the safety assessment, no specific list of adverse events unique to

radiofrequency treatment (such as bile fistula, bleeding, and thermal injury to adjacent organs caused by excessive coagulation of marginal tissues) was provided. It is recommended to supplement a "list of radiofrequency-related adverse events", including symptoms, diagnostic criteria, and grading, to ensure the specificity and completeness of safety data. **Thank you for this important observation. In our previous evaluations of the method, we have not observed any significant adverse events (AEs) or adverse device effects (ADEs) directly related to the radiofrequency device or its method of application. However, we acknowledge the importance of specifying potential radiofrequency-related complications to ensure the completeness and specificity of safety data.**

To assist investigators in identifying and reporting relevant events, we have defined the following as serious surgery-related complications, which may also encompass potential device-related adverse events:

Commented [PSV1]: habría que incluir éstos como en una anexo 1 o algo asi. y hacer una ref. en el texto

- **Postoperative bleeding requiring transfusion of more than 4 units of erythrocyte concentrates**
- **Severe sepsis, as defined by the American College of Chest Physicians/Society of Critical Care Medicine**
- **Subphrenic or perihepatic abscess requiring drainage**
- **Re-laparotomy**
- **Biliary fistula persisting for more than 10 days with output >10 ml/day**
- **Transient liver failure with bilirubin >10 mg/dL lasting more than 3 days**
- **Renal failure requiring dialysis**
- **Respiratory failure requiring mechanical ventilation**
- **Patient death**

In addition, we will supplement the protocol with a dedicated list of potential radiofrequency-related adverse events, including suggested symptoms, diagnostic criteria, and severity grading, to further support accurate reporting and safety monitoring.

Please let us know if additional details are needed.

VERSION 2 - REVIEW

**Reviewer**	**2**
**Name**	**Li, Jianwei**
**Affiliation**	**Chinese Research Group for Minimally Invasive Anatomical Liver Resection**
**Date**	**13-Sep-2025**
**COI**	

The author has addressed all the revision points thoroughly, and the revised manuscript is now clear, logically structured, and suitable for publication.
